# Systematics, Phytochemistry, Biological Activities and Health Promoting Effects of the Plants from the Subfamily Bombacoideae (Family Malvaceae)

**DOI:** 10.3390/plants10040651

**Published:** 2021-03-29

**Authors:** Gitishree Das, Han-Seung Shin, Sanjoy Singh Ningthoujam, Anupam Das Talukdar, Hrishikesh Upadhyaya, Rosa Tundis, Swagat Kumar Das, Jayanta Kumar Patra

**Affiliations:** 1 Research Institute of Biotechnology & Medical Converged Science, Dongguk University-Seoul, Goyangsi 10326, Korea; gdas@dongguk.edu; 2 Department of Food Science & Biotechnology, Dongguk University-Seoul, Goyangsi 10326, Korea; spartan@dongguk.edu; 3 Department of Botany, Ghanapriya Women’s College, Dhanamanjuri University, Imphal 795001, India; ningthouja@hotmail.com; 4 Department of Life Science and Bioinformatics, Assam University, Silchar, Assam 788011, India; anupam@bioinfoaus.ac.in; 5 Department of Botany, Cotton University, Guwahati, Assam 781001, India; hkupbl_au@rediffmail.com; 6 Department of Pharmacy, Health and Nutritional Sciences, University of Calabria, Via P. Bucci, 87036 Rende, Italy; rosa.tundis@unical.it; 7 Department of Biotechnology, College of Engineering and Technology, Biju Patnaik University of Technology, Bhubaneswar, Odisha 751003, India; das.swagat@gmail.com

**Keywords:** bombacoideae, pharmacology, phytochemical ingredients, bioactive compounds, medicine

## Abstract

Plants belonging to the subfamily Bombacoideae (family Malvaceae) consist of about 304 species, many of them having high economical and medicinal properties. In the past, this plant group was put under Bombacaceae; however, modern molecular and phytochemical findings supported the group as a subfamily of Malvaceae. A detailed search on the number of publications related to the Bombacoideae subfamily was carried out in databases like PubMed and Science Direct using various keywords. Most of the plants in the group are perennial tall trees usually with swollen tree trunks, brightly colored flowers, and large branches. Various plant parts ranging from leaves to seeds to stems of several species are also used as food and fibers in many countries. Members of Bombacoides are used as ornamentals and economic utilities, various plants are used in traditional medication systems for their anti-inflammatory, astringent, stimulant, antipyretic, microbial, analgesic, and diuretic effects. Several phytochemicals, both polar and non-polar compounds, have been detected in this plant group supporting evidence of their medicinal and nutritional uses. The present review provides comprehensive taxonomic, ethno-pharmacological, economic, food and phytochemical properties of the subfamily Bombacoideae.

## 1. Introduction

The plant group Bombacoideae is a subfamily of Malvaceae (kapok, cotton family). The subfamily contains about 304 species, most of them with high economical and medicinal values. Considering their importance, some of the plants are given special cultural status. For instance, the *Ceiba pentandra* tree is the national tree of Guatemala. Among the Mayan and Aztec civilizations in the Meso-America, the *Ceiba* species is considered as a sacred “World Tree”. The Indian kapok tree, *Bombax ceiba,* is worshipped by the Hindu community in North India as a nakshatra tree and home of the female spirits Yakshi [1]. There is West African belief that the first human was born from the trunk of a baobab tree (*Adansonia* spp.) and these plants are regarded as the “Tree of Life”. Many plants of the Bombacoideae are valued as ornamentals in various parts of the world because of their large branches and brightly colored flowers [2]. Moreover, many genera of this subfamily are known for producing fibers, timber, fruits, and vegetables, thereby, regarded as one of the important economic and commercial plant groups.

This group was previously recognized as a distinct family, Bombacaceae, based on the type genus *Bombax* by some traditional taxonomists. From the days of the natural system to the present days of phyletic classification, the status of this plant group is continuously debated. Apart from that, the number of genera under this family varied from one classification system to another. There are various arguments in favor of a distinct family or whether to subsume under a subfamily or tribe. The study of palyno-morphological characteristics supported the justification of separating Bombacaceae from Sterculiaceae, Malvaceae, and Tiliaceae [3]. Most of the traditional methodical educations related to the subfamily Bombacoideae are on the basis of the characteristics of the flower, especially the androecium [4]. Recently, morphological, anatomical, palynological, phytochemical, and molecular phylogenetic analyses have shown that separation of Bombacaceae from its related groups viz. Malvaceae, Tiliaceae, and Sterculiaceae is inconsistent [5]. This plant group includes several plants, which are used for medicinal and economic utilities. A detailed search on the number of publications related to the Bombacoideae subfamily was carried out in databases like PubMed and Science Direct and it was found that, as per the PubMed database, around 20 articles have been published during the years 1999–2020 and among them, 12 articles are full texts (https://pubmed.ncbi.nlm.nih.gov/?term=Bombacoideae; accessed on 12 October 2020). Interestingly, from a total of 20 articles, 16 were published during the last 10 years (2010–2020). Similarly, the Science Direct databases show a total of 53 articles were published during the years 1999–2020, of which, 42 are research articles, 2 are review articles, 4 book chapters, 1 short communication, 2 encyclopedia, and 2 others (https://www.sciencedirect.com/search?qs=Bombacoideae&show=100; accessed on 12 October 2020). Considering the importance, the authors attempted to extensively review the taxonomic, phytochemical, and medicinal utilities of the members of the subfamily Bombacoideae.

## 2. Taxonomy of the Subfamily Bombacoideae

**Figure 1:**
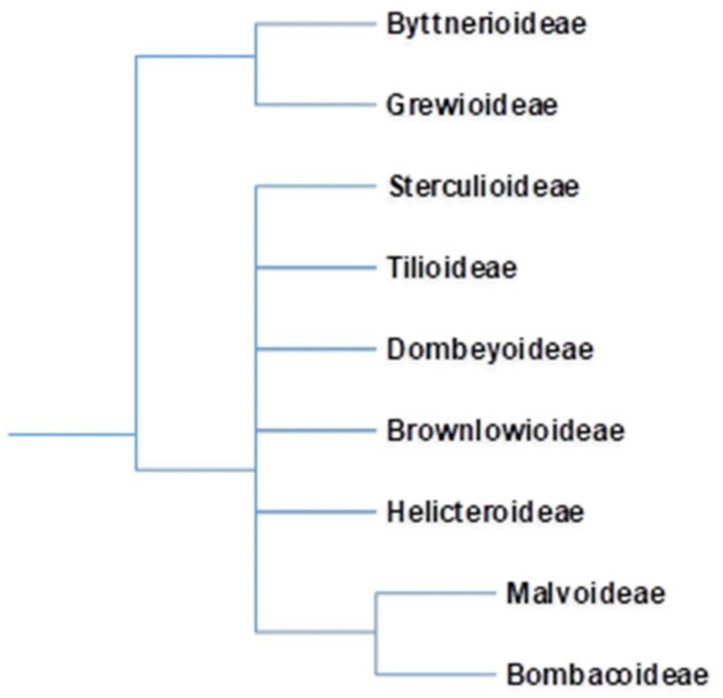
In the Angiosperm Phylogeny Group (APG) classification, erstwhile family Bombacaceae is allocated as subfamily Bombacoideae of the family Malvaceae. Cladogram of the Malvaceae is after Bayer et.al. 1999 and online version of APG (http://www.mobot.org/MOBOT/research/APweb; accessed on 10 January 2021).

**Figure 2:**
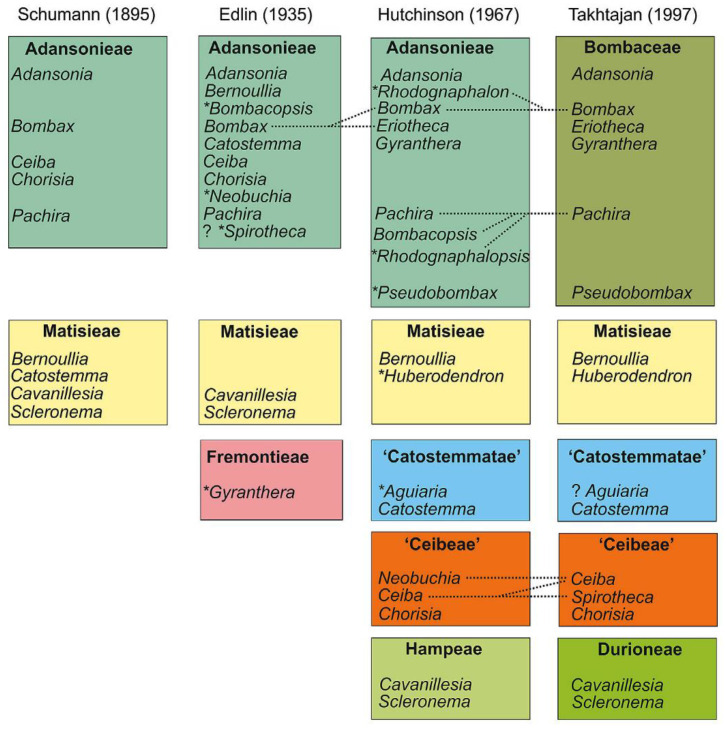
Taxonomic location of Bombacoideae as per different systems of classification. Dotted lines specify the changes in the genus limitation and the genera, which is described after the previous action, and are specified by a symbol (*), while the citation marks represent the tribes which are not validly published. Reproduced with permission from Carvalho-Sobrinho et al. [4] (originally Figure 1).

The advent of new taxonomical tools has revolutionized taxonomical circumscriptions. Morphological and molecular analyses revealed that Bombacaceae is not a monophyletic group. Furthermore, families such as Tiliaceae, Sterculiaceae, and Malvaceae are largely nonmonophyletic. Singh [6] considered that traditional distinctions amongst these four families are random and unpredictable and fusion of four would form a monophyletic group. Bayer et al. [7], grouped these four families together into Malvaceae considering their common characteristics and assumed them to be monophyletic. The Malvaceae sensu lato is characterized by apomorphic inflorescence, presence of bicolor unit, 3-bracted cyme, and trimerous epicalyx. Bombacaceae was distributed into two subfamilies, Bombacoideae and Helicteroideae, within the family Malvaceae. The confinement of Bombacoideae and Malvoideae is still under controversy, as the former appears to be paraphyletic without the latter [8]. Most of the plants are included in the subfamily Bombacoideae. At present, Bombacoideae is one of the clades in the family Malvaceae (Figure 1). The taxonomic location of Bombacoideae as per different systems of classification is shown in Figure 2 [4].

As a consequence of changes in circumscription and status of Bombacoideae, has led to the inclusion of 22 genera comprised of 120 species under this subfamily mainly distributed in the tropical regions. The Angiosperm Phylogeny Group (APG) IV Classification listed 24 genera in this subfamily. However, the classification by Maarten et.al. listed 27 genera under Bombacoideae [9]. This classification includes genera like *Camptostemon, Lagunaria,* and *Uladendron* in the Bombacoideae sub family. Molecular phylogenetic analysis established on nuclear (ETS, ITS) and plastid genes (matK, trnL-trnF, trnS-trnG) revealed that there are three key lineages noticeable by the kapok clade, seed, or fruit traits—the winged seed clade, and the spongy endocarp clade [4]. Such studies established the monophyly of the core Bombacoideae subfamily and the entire genera without *Pachira*. The monospecific *Septotheca* falls outside the core Bombacoideae in many studies [4,10].

## 3. Habitat, Distribution, and Characteristics of the Subfamily Bombacoideae

**Figure 3:**
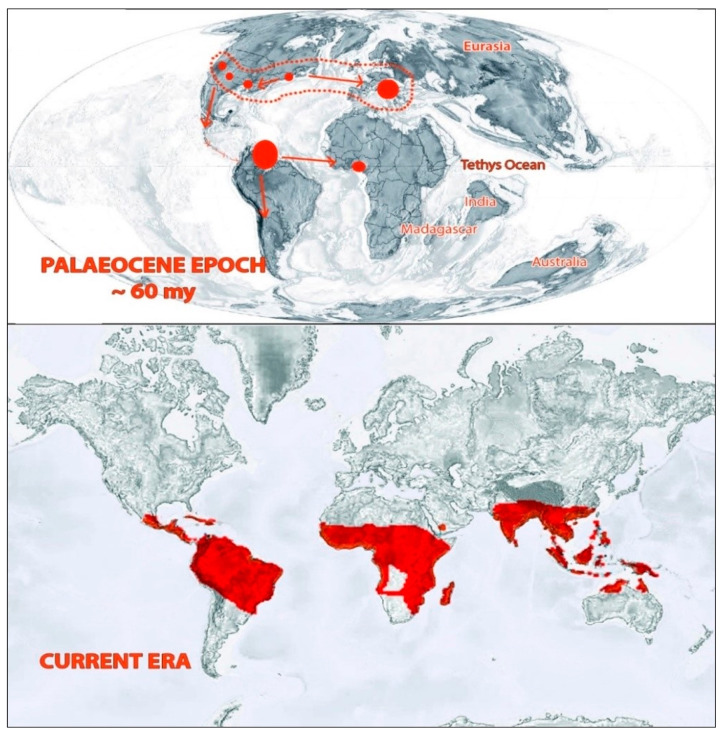
Distribution of Bombacoideae in past and present eras. Adapted from Krutzsch, [20], Zizka et.al. [11], and Angiosperm Phylogeny website version 14 (www.mobot.org/MOBOT/research/APweb; accessed on 10 January 2021).

Bombacoideae occupies different habitats in various parts of the world (Figure 3) [11]. *Adansonia digitata* is confined to semi-arid, stony, hot, dry, and woodland areas, with low rainfall. This plant favors well-drained soils ranging from clays to sandy soils [12]. However, some other plants favor wet and humid habitats. For instance, *Bombax ceiba* favors humid lowland deciduous forest and is sometimes found near stream banks [13,14]. Species belonging to *Spirotheca* are epiphytic stranglers. Some species are part of mangrove vegetation in the tropical regions, for example, *Pachira aquatica, Camptostemon philippinense*, et cetera. The majority of the species in Bombacoideae prefer rain forest biome and seasonally dry biomes [11]. Several representative plant species belonging to the subfamily Bombacoideae grow in different habitats. *Adansonia digitata* L. grows in the hot, semi-arid region with poorly drained soil [15]. Plants such as *Bombax ceiba* L., *Ceiba pentandra*; and *Gyranthera caribensis* Pittier grow in wet habitats [16]. *Pachira aquatica* Aubl. and *Camptostemon philippinense* (S.Vidal) Becc. grow in mangrove habitats [17,18]. *Spirotheca rivieri* (Decne.) Ulbr. grows in the epiphyte environment [19] and *Ceiba pentandra* grows in the savannah habitat [15].

The early distribution of this plant group can be ascertained from fossil records. There are various arguments for the distribution of Bombacoideae. Croizat (1952) favored the knowledge of an African entrance with Bombacoideae transferring northwards from Antarctica by Madagascar, across into Africa and through the East Indies to Australia [21]. However, the concept cannot be supported by floral evolution and geological shreds of evidence [22]. According to another view based on palyno-morphological characteristics exhibited by the members of this plant group, the subfamily is assumed to have a triphyletic origin—with southern Central America, East Africa, Madagascar, and Southeast Asia as centers of origin [3]. Fossil records of this subfamily mainly belonged to microfossils (classified as belonging to the pollen genus *Bombacacidites*) and some macrofossils [23]. This plant group occurred in the North Tethyan flora and reached tropical regions of South America through Central America during the transition phase between the Cretaceous and Tertiary periods. Then they moved to Central Africa in the Paleocene epoch. During the Pliocene and Pleistocene periods, this group extended its distribution to the Caribbean and Central America. When the tropical flora reduced along with the North Tethys during the Upper Paleogene, the Bombacoideae retreated to North India and reached South East Asia during the Miocene epoch. From there, they expanded to New Guinea and North Australia [20,23]. In the present era, the distribution of the extant species mainly falls in the tropical regions, particularly in Africa, America, and Australia. More than 80% of the species’ richness of this subfamily lies in the Neotropical region [8,11].

There are many reports of native species in Asian countries. Various native species are introduced to other parts of the world through human activities and other influences. The center of origin of the species of this plant group differs according to the genus. Species of this plant group can be categorized into two groups—plants endemic to a certain area and plants widely distributed through introduction. Of the endemic species, *Adansonia suarezensis* H.Perrier and *Adansonia gregorii* F.Muell. are restricted to Madagascar and NW Australia, respectively [24]. Madagascar has many endemic species of *Adansonia* such as *Adansonia fony* Baill., *Adansonia madagascariensis* Baill., *Adansonia za* Baill., and *Adansonia perrieri* Capuron [25,26,27]. Wild regions of the endemic species are as follows: *Adansonia suarezensis*, *Adansonia fony* Baill., *Adansonia madagascariensis* Baill., *Adansonia za* Baill., *Adansonia perrieri* Capuron, and *Adansonia grandidieri* Baill. are the endemic species in the Madagascar region [24]. Similarly, *Adansonia gregorii* F.Muell., *Aguiaria excelsa* Ducke, *Uladendron codesuri* Marc.-Berti, *Gyranthera darienensis* Pittier, *Cavanillesia chicamochae* Fern. Alonso, *Gyranthera caribensis* Pittier, and *Neobuchia paulinae* Urb. are endemic to Australia, Brazil, Venezuela, Panama, Colombia, Venezuela, and Haiti, respectively [4,8,24,28,29,30].

Native regions of various species in this group fall within the tropical region of Africa, America, and Asia. From their native regions, many species have been introduced to other parts of the world. *Adansonia digitata* is amongst the most widely distributed ones covering Asia, Australia, Northern America, and some oceanic islands. Distribution of this plant in the Caribbean and parts of America is through human agencies, where people from West Africa were transported between the sixteenth and nineteenth centuries for sugarcane plantations in the New World countries. In the Indian subcontinent, Arab traders or medieval Muslim rulers who maintained African slave armies mainly introduced this species. However, genetic analyses conducted in Indian populations revealed that the introduction occurred through multiple phases [31]. Most of the species have neotropical distribution, with some species having native ranges in Asia. *Bombax ceiba* has wild distribution in South East Asia and India. The place of origin of some plants is uncertain. The origins of wild areas of *Ceiba pentandra* (L.) Gaertn. are uncertain but now it is distributed throughout tropical regions including Asia [32].

## 4. Characteristics

Plants of Bombacoideae are usually perennial tall trees usually with swollen tree trunks. Trees of wet forests are usually evergreen while those of dry forests are deciduous [33]. Tree trunks may contain parenchymatous water storage tissue or mucilage cells. Pneumatophores are present in the *Camptostemon*, a mangrove genus [8]. Barks are usually thin, often green. Most of the plants of Bombacoideae are characterized by their large size gigantic flowers with brush types [8]. Plants in these groups have a terminal flower and three bracts that exhibit a “bicolor unit”. The first, lowermost bracts remain sterile, however, other bracts subtend cymose partial inflorescences. Flowers are usually subtended by an involucre of bracts. Sepals are usually large and fused and petals are usually fused to the stamen tube [34]. The fruit capsule has a hairy endocarp. Leaves are usually peltately-palmate. Petioles are pulvinate, k connate with or without lobes. Monothecal anthers are present. These characteristics are assumed to have resulted from the splitting of whole stamens. Transitional forms are observed in some plants [8]. Anther walls have 5–7 cells across. Staminodes are usually absent. Pollen may be flattened, triangular in polar view. Seeds are usually large and usually more than two cm long.

Most of the members of this subfamily are trees, especially shrubs, with characteristic two to five carpels, fruit capsules, rarely indehiscent, endocarp usually pubescent, pollen usually without spines, seeds usually glabrous, and exceptionally spinulose [8]. Some plants have a ploidy level other than diploidy. The lowermost chromosome numbers in this group were witnessed in *Bombax insigne* (2n = 18) from India and *Pachira macrocarpa* (2x = 26) from China, while uppermost numbers were documented in *Eriotheca* species (6x = 276) in Brazil [35]. Distinguishing characteristucs of the genera in this subfamily is provided in Table 1.

The status of genera under Bombacoideae might be subjected to change in future revisions. The single species *Chiranthodendron pentadactylon* can be crossed with *Fremontodendron* sp. [8] exhibiting compatible genotypes. *Neobuchia paulinae* is an imperfectly known species that may be included in *Ceiba* [8].

## 5. Phytochemical Configuration of Bombacoideae Subfamily

Phytochemical investigations of Bombacoideae plant species resulted in the extraction and isolation of several classes of secondary metabolites. Among the most studied genera, there are *Adansonia*, *Bombax*, and *Chorisia* [2,37,38]. *Bombax ceiba* (syn. *Bombax malabaricum*, *Bombax malabarica*, *Salmalia malabaricum*, *Gossampinus malabarica*), *Adansonia digitata*, and *Chorisia speciosa* are the most chemically and biologically investigated species.

A wide spectrum of phytochemicals has been identified and has confirmed that this family is a rich source of phytochemicals. Table 2 lists the main alkaloids, anthocyanins, coumarins, flavonoids, lignans and neolignans, sesquiterpenes and sesquiterpene lactones, sterols, tannins, and triterpenes isolated from the Bombacoideae subfamily. Volatiles and fatty acids were also reported (Table 2).

The fruit pulp of *A. digitata* from Mali is characterized by flavonol glycosides and procyanidins as dominant classes of compounds [50]. Tiliroside was identified as a major constituent. *A. digitata* fruits from Nigeria showed hydroxycinnamic acid glycosides, iridoid glycosides, and phenylethanoid glycosides, secondary metabolites not detected in the fruits from Mali [88]. More recently, procyanidins, phenolic acids, and flavonol glycosides were identified in *A. digitata* fruits from Cameroon [89]. In particular, fruit pulp was characterized by the presence of non-flavonoid compounds such as hydroxycinnamic derivatives and flavonoids, mainly flavones, flavanols, proanthocyanidins, and flavonols.

Furthermore, polar compounds identified in leaf extracts consisted of several classes of flavonoids and hydroxycinnamic acids. Leaves from Cameroon [89] exhibited a very similar profile compared to the leaves from Mali [50].

Previously, Tembo et al. [90], quantifying several compounds in fresh *A. digitata* pulp and investigating quantitatively variations of some of these molecules induced by pasteurization and thermal preservation, described a high content of epicatechin, gallic acid, and procyanidin B2 in Malawi *A. digitata* fruits. Nasr et al. [65] isolated two flavonoid glycosides, namely, rhoifolin and tiliroside, in the alcoholic extract of *C. speciosa* leaves from Egypt, together with some sterols and triterpenes. The sesquiterpenes, bombamalin and isohemigossypol-1-methyl ether, and the phenols, 4-hydroxy-3,5-dimethoxybenzoic acid, 3,4,5-trimethoxyphenol-1-(β-xylopyranosyl-(1→2))-β-glucopyranoside, shorealactone, (−)-epicatechin 5-*O*-β-D-xylopyranoside, and 2-C-(β-D-apiofuranosyl-(1→6))-β-D-glucopyranosyl-1,3,6-trihydroxy-7-methoxyxanthone have been isolated from the ethanol extract of *B. malabarica* root bark [73].

**Figure 4:**
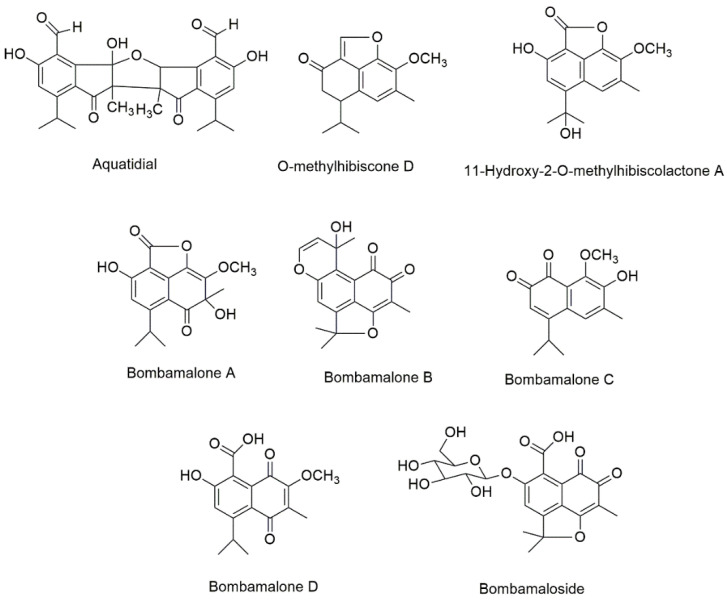
The chemical structures of new isolated compounds from Bombacoideae species.

Five new compounds, namely, bombamaloside and bombamalones A–D (Figure 4), were obtained by Zhang et al. [74] from the H_2_O/acetone (3:7) extract of *B. malabaricum* roots, along with other known constituents such as bombaxquinone B, lacinilene C, isohemigossypol-1-methyl ester, and 2-*O*-methylisohemigossylic acid lactone.

Aquatidial (Figure 4) was previously isolated from a chloroform extracts of *P. aquatica* roots together with the known compounds lupeol, triacontyl *p*-coumarate, and isohemigossypolone [72]. Aquatidial is a new bis-norsesquiterpene with an uncommon skeleton, putatively derived from isohemigossypolone. Two new naphthofuranones, 11-hydroxy-2-*O*-methylhibiscolactone A and *O*-methylhibiscone D (Figure 4), have been extracted from the *P. aquatica* stems [48].

Several volatiles have also been described from some Bombacoideae species. Sulfur compounds (15.3%), benzenoids (7.8%), monoterpene hydrocarbons (0.6%), and oxygenated monoterpenes (0.2%) were identified in the flowers of *A. digitata* [91]. The oil obtained from the flowers of *C. pentandra* showed monoterpene hydrocarbons (34%), sesquiterpene hydrocarbons (26.9%), oxygenated monoterpenes (8.4%), benzenoids (7.8%), and miscellaneous compounds (2%) [91].

The most common fatty acids in the Bombacoideae subfamily are oleic, linoleic, linolenic, stearic, and palmitic acids. The cyclopropenoid fatty acids, malvalic acid and sterculic acid, have been identified in *A. digitata* [92,93,94], *A. fony* [94], and *Bombax oleagineum*, *C. acuminata*, and *C. pentandra* [61]. Recently, the seeds’ *n*-hexane extract of *C. speciosa* from Italy showed linoleic acid (28.22%) and palmitic acid (19.56%) as the most abundant fatty acids [95]. Percentages of 16.15 and 11.11% were found for malvalic acid and sterculic acid, respectively.

Linoleic acid (38.8%), palmitic acid (24.3%), and oleic acid (21.9%) were identified as the dominant fatty acids of *C. pentandra* seed oil from Malaysia [96]. Malvalic and sterculic acids were also identified. A lower percentage of linoleic acid was found in the seed oil of *C. pentandra* from India [97]. Saturated fatty acids and monounsaturated fatty acids were obtained from the seeds of *P. aquatica* by using the Soxhlet apparatus and *n*-hexane as solvent. Palmitic acid and oleic acid were the most abundant with percentages of 49.0 and 18.2%, respectively [98]. Linoleic acid (11.2%) is the only polyunsaturated fatty acid identified.

## 6. Details of the Extraction and Isolation Procedure of Major Compounds from Bombacoideae for Industrial Applications

Different bioactive constituents, mainly terpenes, flavonoids, alkaloids, steroids, and fatty acids, have been isolated from the Bombacoideae subfamily. The extraction technique is the first pivotal step to obtaining active phytochemicals from plants. The choice of extraction procedure would depend mainly on the advantages and disadvantages of the process, including yield, biological activity, environmental friendliness, and safety. The fruit pulp of *A. digitata* revealed the presence of iridoids and phenols by using 70% ethanol as solvent [88]. Proanthocyanidins were obtained as major constituents from the pericarp of *A. digitata* fruits [54] by using a hydroalcoholic solution (methanol/H_2_O 80:20 *v*/*v*). Maceration with 95% ethanol of *B. malabarica* root bark led to the isolation of several sesquiterpenes, triterpenes, phenols, and sterols [73]. Conversely, cadinene sesquiterpenes were extracted from the roots of *B. malabaricum* by using H_2_O/acetone (3:7 *v*/*v*) [74]. *B. malabaricum* flowers extracted by 70% (*v*/*v*) aq. ethanol is characterized by different lignans.

Until now, the most applied extraction technique to isolate phytochemicals from the Bombacoideae subfamily is maceration. Researchers are exploring other extraction procedures using less energy and less solvent while producing higher yields and that are more environmentally friendly. Some advanced methods (i.e., pressurized and accelerated fluid extraction, supercritical extraction) have demonstrated to be useful in mediating related extraction difficulties along with increased extraction yields. Two of the most commonly employed extraction techniques of flavonoids are microwave- (MAE) and ultrasound-assisted extraction (UAE). High extraction efficiency and less destruction of the active constituents are the many advantages of UAE [99,100,101]. Nevertheless, MAE is preferred over UAE because MAE has been shown to increase the mass transfusion through the solid matrix, faster mixing of the extraction solvent thus preserving the highest possible driving forces, and ensuring the highest quality and quantity of the extracted constituents. Indeed, several works have proven that MAE allows for great extraction yields, a reduction of the volumes of solvents used, and a reduction of the extraction times [99,100]. MAE has been applied to extract flavonoids, tanshinones, coumarins, and terpenes [101]. These characteristics along with the simplicity of operation would position MAE as a valuable and suitable technology for industries with the growing demand for increased productivity and efficiency. However, until now little progress has been described for the MAE application to Bombacoideae species. Surely, taking into account all the MAE features, in the future, it will be possible to optimize the process by exploiting the opportunity to apply this innovative extraction method to the study of species belonging to the Bombacoideae family.

## 7. Application in Food/Use as Food

From ancient periods until today, many plants of the Bombacoideae have been used as food in various corners of the world. Parts used may range from leaves, seeds, tuberous roots to stem, flowers, et cetera. There are various variations in the use of food according to genera and cultures associated. Native African populations commonly use fruits of *Adansonia digitata* as famine food to make sauces, decoctions, and refreshing beverages [102]. The leaves, seeds, and pulp of the fruit of this plant are all edible. Lim (2012) reported the use of young leaves, seeds, fruit pulp, and tuberous roots of *Adansonia gregorii* F. Muell as food. Along with *Adansonia* spp, *Ceiba pentandra* is another one of the plant foods common to West Africa. Leaves of this plant are cooked in the form of slurry sauce [103]. The utilization as food for this plant group is not restricted to Africa but observed in other parts of tropical countries. In Central and South America, flowers and tender leaves of *Pachira aquatica*, a wetland tree, are cooked and used as vegetables [15]. Young roots of *Bombax ceiba* are eaten raw or roasted in Cambodia. The cuipo tree (*Cavanillesia platanifolia*), growing in Central America, is used by the natives for getting water. To collect water, a piece of the root is cut and the bark is removed on one end after keeping the root horizontal. When the clean end of the root is lowered, the water drains out through the cut end [104].

The use and preparation of food from the members of Bombacoideae dates back to time immemorial. For instance, in South America, from the ancient pre-Colombian period [105], flowers of *Quararibea funebris* were used as an additive to chocolate drinks. Ancient Mayans used the sap from *Pseudobombax ellipticum* to make an intoxicating drink by fermentation. This drink was likely used in religious ceremonies such as sacrifice and self-mutilation [33]. The use of various members of Bombacoides as fruits, vegetables, and other forms are highlighted in Table 3.

## 8. Traditional and Economic Uses

Various members of Bombacoideae are used as fiber and other utilities and some are also used as ornamental plants. *Adansonia digitata* is a multipurpose plant with various economic and social values [106]. In African countries, *Adansonia digitata* is very popular and reported to have more than three hundred traditional uses [102]. *Ceiba* Mill. is now popular throughout the tropical regions for ornamental landscaping [114]. Many species of the genus *Ceiba* were sacred to the Mayan civilization as depicted in ancient ceramics because of their cultural importance [33].

Many Bombacoideae species are economically important. Some species are collected for their wood that is soft and can easily be carved into canoes and other useful products. One popular wood is balsa wood obtained from the *Ochroma pyramidale* [16] and other species were widely used for making dugout canoes in ancient South America. Ancient Peruvians are believed to have used legendary Kon-Tiki rafts made from balsa wood to navigate across the Pacific Ocean and settle in the Polynesian islands [115]. The silky cotton-like fluff (kapok) present in the seed pods of *Ceiba pentandra* is used for stuffing pillows, bedding, and soft toys in various parts [116]. Silk hair present in seeds of *Bombax ceiba* are used in India from time immemorial for stuffing cushions, mattresses, pillows, and making clothes [117]. Various traditional and economic uses of the members of this subfamily are summarized in Table 4.

## 9. Ethnopharmacology

In various tropical countries, plants of Bombacoideae are used in traditional medicine mainly for pharmacological properties like anti-inflammatory, astringent, antimicrobial, stimulant, antipyretic, analgesic, and diuretic [2]. For instance, various parts of *Bombax ceiba* such as the stem bark, flowers, fruits, seeds, leaves, and root of young plants, are traditionally used as remedy in South India [108]. Its main therapeutic applications include diabetes, urinogenital disorders, gastrointestinal and skin diseases, gynecological, and general debility [129]. Another important plant from this subfamily in the Indian ayurvedic system is *Ceiba pentandra* known as Sweta Salmali for its acrid, bitter, thermogenic, diuretic, and purgative properties. The known pharmacological activities of *Ceiba pentandra* include hepatoprotective, antidiabetic, antipyretic, laxative, and anti-inflammatory [130]. *Adansonia digitata* is one of the most studied species for its therapeutic properties against antipyretic, diarrhea, dysentery, and as a substitute for cinchona in traditional medicinal preparations [105]. Different species under Bombacoideae having reported ethnopharmacological uses are summarized in Table 5.

## 10. Pharmacological Potential of Bombacoideae

**Figure 5:**
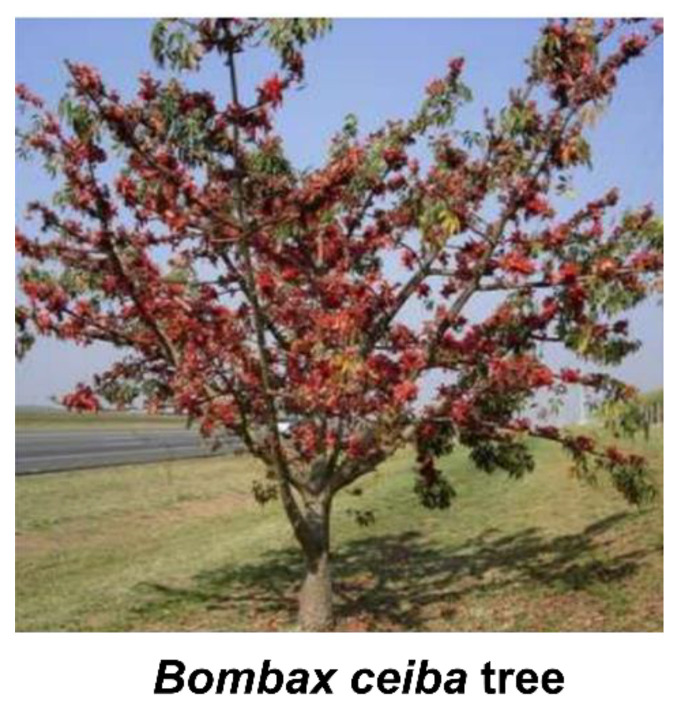
Photo of representative plant species from the Bombacoideae subfamily. Reproduced under Creative Commons Attribution-Non-Commercial 4.0 International License (https://creativecommons.org/licenses/by-nc/4.0/; accessed on 27 February 2021) from Rameshwar et al. [136] (originally Figure 1).

The different species, viz. *Adansonia digitata*, *Bombax ceiba*, *B. malabaricum*, and *Ceiba pentandra* of the Bombacoideae family [136,146], were reported for their various pharmacological potentials, which are summarized in the following section (Table 6; Figure 5).

### 10.1. Antioxidant Properties

*Adansonia digitata* L.

The methanolic fruit pulp and leaf extracts of *A. digitata* exhibited in vitro antioxidant activities as studied by 2,2-diphenyl-1-picryl-hydrazyl-hydrate (DPPH), 2,2-azinobis—(3-ethylbenzothiazoline-6-sulfonate) (ABTS), ferric reducing antioxidant power (FRAP), β-carotene bleaching test, superoxide-scavenging assays [50,150]. The methanol extracts of leaf, seed, bark, fruit wall, and floral extracts of *A. digitata* were reported for their DPPH scavenging potential [147]. The DPPH scavenging activity was highest in seed extract (27.69%) and lowest in fruit wall (20.69%) extract. The methanolic leaf extract of *A. digitata* could maintain the antioxidant status of the streptozotocin (STZ) induced diabetic rats by normalizing the elevated levels of reduced glutathione (GSH) superoxide dismutase (SOD), and catalase (CAT) [148]. The ethanolic leaf, bark, and fruit extracts of *A. digitata* could scavenge the DPPH free radicals with percentages of inhibition of 13.4, 29.23, and 39.21%, respectively [149].

*Bombax ceiba* L.

The methanolic root extract of *Bombax ceiba* could scavenge DPPH radicals, lipid peroxidation, and ascorbyl radicals with an EC_50_ value of 87 µg/mL. The extract also inhibited lipid peroxidation in rat-liver microsome induced by ascorbyl and peroxynitrite radicals with IC_50_ values of 141 µg/mL and 115 µg/mL, respectively [192]. In another study, the methanol root extract of *B. ceiba* scavenged DPPH radical with an EC_50_ value of 15.07 µg. The extract also could reduce the Fe^3+^ to Fe^2+^ in a dose-dependent manner with the maximum activity at 500 µg. The study also demonstrated that the administration of 3 g root powder could raise the antioxidant status in the human volunteer. The antioxidant activity properties of the root extract are attributed to their high phenolic and tannin contents [152]. The aqueous soluble partition (AQSF) of the methanolic root extracts of *B. ceiba* scavenged DPPH radical with an IC_50_ value of 3.33 μg/mL [153]. Further, the methanol and petroleum ether root extract of *B. ceiba* was reported to scavenge DPPH radical with IC_50_ values of 144.77 and 214.83 μg/mL [155]. The methanolic stem bark extract of *B. ceiba* exhibited antiradical activity with EC_50_ values of 18.78, 23.62, and 139.4 μg/mL for nitric oxide, DPPH, and reducing power activity assay, respectively [193]. Similarly, Hossain et al. [154] reported the antioxidant activity of methanolic root extract of *B. ceiba* by DPPH scavenging assay (IC_50_ value of 58.6 μg/mL). Gandhare et al. [156] reported that aqueous and ethanolic extracts of the *B. ceiba* bark exhibited DPPH, ABTS, nitric oxide, and superoxide radical scavenging activity along with total antioxidant activity. Besides the extract also inhibited lipid peroxidation and reduced ferric ions. The IC_50_ values of aqueous extracts of *B. ceiba* varied between 85.71 and 102.45 µg/mL, and for ethanolic extract, it varied between 85.48 and 103.4 µg/mL. Komati et al. [163] reported that aqueous methanol extract of *B. ceiba* calyx reduced the level of reactive oxygen species (ROS), NADPH oxidase (NOX), and thereby lowered the mitochondrial dysfunction in methylglyoxal induced protein glycation. Further, in HEK-293 cells, Mn and Cu/Zn-superoxide dismutase and glutathione reductase antioxidant enzymes levels were improved. The whole plant methanolic extract of *B. ceiba* scavenged DPPH radical with an IC_50_ value of 68 µg/mL [158]. The petroleum ether (PE) of *B. ceiba* flowers exhibited DPPH and Fe-chelating activities with IC_50_ values of 37.6 and 33.5 μg/mL and diethyl ether extracts (DE) exhibited beta-carotene bleaching test with an IC_50_ value of 58.3 μg/mL. The antioxidant properties of *B. ceiba* flower extracts are attributed to the presence of beta-sitosterol and some fatty acids [80]. Similarly, another study reported that aqueous flower extracts of *B. ceiba* could scavenge DPPH radicals with an IC_50_ value of 50.21 μg/mL [159]. The aqueous flower extracts of *B. ceiba* exhibited antioxidant activities against DPPH, hydroxyl, hydrogen peroxide, and ferric ion reducing antioxidant power (FRAP) activity with IC_50_ values of 1.70 mg/mL, 4.20 mg/mL, 3.51 mg/mL, and 2.15 mg/mL, respectively [160]. The hexane, benzene, chloroform, ethyl acetate, acetone, methanol, and ethanol extracts prepared from methanolic flower extract of *B. ceiba* exhibited DPPH scavenging activity [161]. The hexane, chloroform, and methanolic extracts prepared from dried powder extracts of *B. ceiba* flower exhibited antioxidant activity in terms of FRAP, DPPH, and reducing power assay [162].

*Bombax malabaricum* DC.

The n-hexane and methanol flower extracts of *B. malabaricum* scavenged DPPH radicals over a concentration range of 0.55–0.0343 mg/mL and 0.5–0.0312 mg/mL, respectively. The maximum DPPH scavenging was observed in the range of 0.55–0.5 mg/mL for both extracts [49]. The antioxidant potential of flower extract was attributed to the presence of bioactive constituent, viz. apigenin, cosmetin, xanthomicrol, saponarin, vicenin 2, isovitexin, and linarin. Similarly, in another study, the aqueous, acetone, and ethanol flower extracts of *B. malabaricum* flowers showed DPPH radical-scavenging properties along with Oxygen radical absorbance capacity (ORAC), reducing power, and liposome peroxidation inhibition activities [151].

*Ceiba pentandra* L.

The different stem bark extracts of *C. pentandra* such as decoction, maceration, and methanol scavenged DPPH radical with IC_50_ values of 87.84, 54.77, and 6.15 µg/mL, respectively. The extracts also restrained the H_2_O_2_-induced hemolysis and lipid peroxidation [165]. The Soxhlet seed oil extracts at 100 mg/mL concentration of *C. pentandra* exhibited DPPH, and OH radical scavenging along with FRAP, reducing power activities by 47.65%, 39.69%, and 309 FRAP units, and 20.52 μg of ascorbic acid equivalent, respectively [193]. The in vitro antioxidant evaluation of *C. pentandra* ethanol leaf extract demonstrated that the extract could scavenge DPPH, nitric oxide, and hydroxyl radicals with IC_50_ values of 27.4, 24.45, and 51.65 µg/mL, respectively. The Gas chromatography-mass spectrometry (GC-MS) study revealed the presence of 9 compounds, amongst which, hexadecanoic acid was found to be the most prominent compound [167]. In another study, Fitria et al. [166] demonstrated that a compound vavain or 5, 3′-dihydroxy-7, 4 ′, 5′- trimethoxyisoflavone isolated from the ethyl acetate fraction of stem bark of *C. pentandra* could scavenge DPPH radical with IC_50_ value of 81.66 µg/mL. However, the ethyl acetate extract of the aerial part of *C*. *pentandra* scavenged the DPPH radicals with an IC_50_ value of 0.0716 mg/mL [184]. The aqueous and methanol stem bark extracts of *C. pentandra* inhibited superoxide (O_2_^•^^−^) (IC_50_ values of 51.81 and 34.26 μg/mL), hydrogen peroxide (44.84 and 1.78 μg/mL), and protein oxidation induced by H_2_O_2_ (120.60 and 140.40 μg/mL) [168].

### 10.2. Anti-Inflammatory Activity

*Adansonia digitata* L.

The methanol leaf extracts of *A. digitata* reduced iNOS and NF-*k*B expression in LPS-stimulated RAW264.7, thereby showing its anti-inflammatory potential [181]. The extract could inhibit NO production with an IC_50_ value of 28.6 µg/mL. Similarly, the Dimethyl sulfoxide (DMSO) fruit pulp and aqueous leaf extract of *A. digitata* inhibited expressions of proinflammatory cytokine IL-8 [182]. The leaf extract (70 µg/mL) exhibited better anti-inflammatory activity compared to pulp extract (247 µg/mL).

*Bombax ceiba* L.

The petroleum ether, ethanol, and aqueous bark extracts of *B.* *ceiba* at 1000 µg/mL concentration exhibited anti-inflammatory potential by stabilizing the Human red blood cell (HRBC) membrane. Amongst the different solvent extracts, better anti-inflammatory activity is shown by ethanol extract followed by aqueous and petroleum ether extract [183].

*Ceiba pentandra* (L.) Gaertn

The ethyl acetate extracts of aerial parts of *C*. *pentandra* upon oral administration at 400 mg/kg dose could inhibit methotrexate (MTX)-initiated apoptotic and inflammatory cascades. The extract could improve the architecture of histopathological changes observed in the renal tissue of MTX-induced nephrotoxic rats [184].

### 10.3. Antimicrobial Activity

*Adansonia digitata* L.

The methanolic and ethanolic leaf and stem bark extracts of *A. digitata* inhibited the growth of *S. aureus* and *E. coli* at different concentrations, viz, 100, 200, 500, and 1000 mg/mL with a minimum bactericidal concentration (MIC) at 100 mg/mL [169].

*Bombax ceiba* L.

The methanolic stem bark extract of *B. ceiba* could inhibit the growth of both Gram-negative (*Escherichia coli, Pseudomonas aeruginosa*, and *Salmonella typhi)* and Gram-positive bacteria (*Bacillus subtilis, Staphylococcus aureus)* dose-dependently. The order of sensitivity from highest to lowest was *S. aureus > E. coli > P. aeruginosa > B. subtilis > S. typhi* [157]. The methanolic flower extract of *B. ceiba* exhibited antibacterial activity against *Klebsiella pneumonia, E. coli, P. aeruginosa* (Gram-negative), and *S. aureus, B. subtilis* (Gram-positive) bacteria with the MIC value ranging between 3.125 and 12.500 μg/mL [161]. The methanol, dichloromethane, and PE extracts of *B. ceiba* roots exhibited mild to moderate antibacterial activity against different bacterial strains including *Sarcina lutea, Bacillus megaterium, B. subtilis, S. aureus, B. cereus, P. aeruginosa, Salmonella typhi, E. coli, Vibrio mimicus, Shigella boydii,* and *S. dysenteriae* with a 7–13 mm zone of inhibition [155].

*Bombax malabaricum* DC.

The n-hexane and methanol extracts (at 100 µg/mL) of *B. malabaricum* demonstrated antimicrobial activities against Gram-positive (*E. coli, Neisseria gonorrhoeae, P. aeruginosa*), Gram-negative (*S. aureus, B. subtilis, Streptococcus faecalis*) bacteria and fungi (*Aspergillus niger, A. flavus Candida albicans*). Of the two extracts, the methanol extract showed better activity against all the studied bacterial strains and *C. albicans*. Further, only the methanol extract exhibited moderate activities against *A. niger* and *A. flavus* [49].

*Ceiba pentandra* (L.) Gaertn

The ethyl acetate fraction of leaf and bark of *C. pentandra* showed antimicrobial activity against E. coli, Salmonella typhi, B. subtilis, Kleibsiella pneumonia, and *S. aureus* [170]. Similarly, aqueous, methanol, ethanol, and acetone seed extracts of *C. pentandra* exhibited antimicrobial activity against *E. coli, S. aureus, K. pneumonia, Enterobacter aerogenes, P. aeruginosa, Salmonella typhimurium, S.typhi, Staphylococcus epidermidis*, and *Proteus vulgaris* [171]. Another study revealed that ethanol leaf extract of *C. pentandra* dose-dependently inhibits antibacterial activity against *E. coli* and *S. aureus* [167].

### 10.4. Anticancer and Cytotoxicity Activity

*Adansonia digitata* L.

The seed and pulp extracts of *A. digitata* (at 10, 100, and 500 µg/mL) exhibited anticancer activity against MCF- 7 (breast cancer cell), Hep-G2 (liver cancer cell), and COLO-205 (colon cancer cell) in a dose dependent manner [172]. The results of the MTT study revealed that the inhibition ranges between 22.57 and 29.96% for MCF-7 cell line; 25.85 and 37.81% for Hep-G2 cell line, and 20.75 and 27.34% for COLO-205 cell line. The dichloromethane and methanolic leaf extracts of *A. digitata* demonstrated cytotoxic activity against human breast development cell lines BT474 evaluated by MTT assay. The methanol leaves of the plant exhibited moderate cytotoxic activity (56%) against the BT474 cell line with IC_50_ values of 15.3 ± 0.4 µg/mL [185].

*Bombax ceiba* L.

The diethyl ether and light petroleum ether extracts of *B. ceiba* flowers exhibited antiproliferative activity against human renal adenocarcinoma cell (ACHN) with respective IC_50_ values of 53.2 and 45.5 μg/mL. The antiproliferative properties were attributed to the presence of beta-sitosterol and some fatty acids in *B. ceiba* flowers [80]. The brine shrimp lethality bioassay revealed that the petroleum ether, dichloromethane, and methanol extracts of *B. ceiba* roots exhibited cytotoxic effect with LC_50_ values of 22.58, 37.72, and 70.72 μg/mL, respectively [155].

*Ceiba pentandra* (L.) Gaertn

The petroleum and acetone stem bark extracts of *C. pentandra* at 15 and 30 mg/kg doses could reduce tumor weight by >50% and tumor volume on the 30th day in Dalton’s lymphoma ascites (DLA) model [173]. Similarly, both these extracts of *C. pentandra* exhibited cytotoxic effects against Ehrlich ascites carcinoma (EAC) cells as evaluated by trypan blue assay [173]. At 15 mg/kg doses, both the extracts showed improvement in mean survival time and decline in tumor induced increase in body weight. Further, the petroleum ether, benzene, chloroform, acetone, and ethanolic extract of this plant demonstrated cytotoxicity in a concentration dependent manner after 3 h of incubation with EAC cells with EC_50_ values of 53.30, 70.58, 250.48, 67.30, and 56.11 µg/mL, respectively.

### 10.5. Hepatoprotective Activity

*Adansonia digitata* L.

The aqueous fruit pulp extract of *A. digitata* showed hepatoprotective potential in carbon-tetrachloride (CCL_4_) -induced hepatotoxic rat models as significant reductions in serum aspartate aminotransferase (AST), alanine aminotransferase (ALT), alkaline phosphatize (ALP), and bilirubin levels were observed in extract-treated hepatotoxic rats [186,187]. The liver protection potential could be attributed to the presence of triterpenoids, β-sitosterol, β-amyrin palmitate, and α-amyrin or without, and ursolic acid in the fruit pulp [188]. The methanolic fruit pulp extract of *A. digitata* exhibited hepatoprotective potential in paracetamol-induced hepatotoxic rat models. The disturbances in the liver function such as ALT, AST, ALP, total bilirubin, and total protein measurements of the hepatotoxic rats were normalized due to the administration of paracetamol [189].

*Bombax ceiba* L.

The hepatoprotective property of aqueous [159] and methanolic [190] flower extracts of *B. ceiba* was studied in CCl_4_-induced hepatotoxic rats. Treatment with extracts decreased elevated levels of glutamate oxaloacetate transaminase (SGOT), glutamic pyruvic transaminase (SGPT), alkaline phosphatize (ALP), bilirubin, triglycerides, and total protein. Treatment with the extract further attenuated the damage caused to the liver as seen by histological studies. The young roots of *B. ceiba* exhibited hepatoprotective activities in alloxan induced diabetic mice. Administration of ethanolic root extracts at 400 mg/kg decreased the hepatotoxicity in diabetic mice by reducing the elevated levels of SGOT and SGPT [176].

*Ceiba pentandra* (L.) Gaertn

The ethyl acetate fraction of methanolic stem bark extract of *C. pentandra* exhibited a hepatoprotective effect against paracetamol-induced hepatotoxic rats by reducing the serum enzyme levels of SGOT, SGPT, ALP, and total bilirubin content [191].

### 10.6. Antidiabetic Activity

*Adansonia digitata* L.

The methanolic fruit pulp and leaf extracts of *A. digitata* exhibited in vitro antidiabetic activities by inhibiting the digestive enzyme α-glucosidase dose-dependently [50]. The IC_50_ values of the fruit extracts ranged between 1.71 *±* 0.23 and 2.39 *±* 0.22 µg/mL while the leaf extract had an IC_50_ value of 1.71 *±* 0.23 µg/mL. Similarly, the methanolic leaf extract of this plant inhibited α-amylase, α-glucosidase, and aldolase reductase [150]. The antidiabetic potency of the extracts may be attributed to the presence of catechin, epicatechin, rutin, quercitrin, quercetin, kaempferol, luteolin (flavonoids), gallic, chlorogenic, caffeic, and ellagic acids (phenolic acids). The methanolic leaf extract of *A. digitata* reduced the elevated blood glucose, glycosylated hemoglobin levels in streptozotocin (STZ) induced diabetic rats [148].

*Bombax ceiba* L.

The dichloromethane, ethanol, and aqueous thalamus and flower extracts of *B. ceiba* were reported for their antidiabetic properties in terms of their capacity to inhibit alpha-amylase and alpha-glucosidase enzymes under in vitro condition. The corresponding IC_50_ values for alpha-amylase inhibition activities for thalamus were 36.22 µg/mL (dichloromethane extract), 35.32 µg/mL (ethanolic extract), and 31.31 µg/mL (aqueous extract) and for flowers, 38.13 µg/mL (dichloromethane extract), 35.23 µg/mL (ethanolic extract), and 33.00 µg/mL (aqueous extract) [174]. The *n*-hexane fraction of sepals [175] and ethanolic leaf extracts [177] of *B. ceiba* exhibited antidiabetic activities in STZ-induced diabetic rats. The *n*-hexane fraction at 0.1 gm/kg bw, b.d. dose reduced the fasting blood sugar level and restored the levels of serum insulin, Hb, and glycated hemoglobin in diabetic rats. Histological studies of also showed marked improvement in diminution in the area of the islets of Langerhans of pancreases in diabetic rats treated with the plant extracts [175]. Similarly, the leaf extract of *B. ceiba* (at 70, 140, and 280 mg/kg doses) decreased the fasting blood glucose, glycosylated hemoglobin, and increased the oral glucose tolerance in the STZ-induced diabetic rats. The antidiabetic property may be attributed to the antioxidant activity and protecting pancreatic β-cells of the extract [177]. The young roots of *B. ceiba* exhibited antidiabetic activities in alloxan-induced diabetic mice. Administration of ethanolic root extracts at 400 mg/kg decreased blood glucose levels in diabetic mice as compared to untreated diabetic mice at different time points (0–24 h). [176]. However, at 600 mg/kg dose the extract could significantly decrease elevated levels of blood glucose in diabetic rats [178].

*Ceiba pentandra* (L.) Gaertn

The aqueous stem bark extracts of *C. pentandra* exhibited antihyperglycemic, insulin-sensitizing potential, and cardioprotective effects in dexamethasone-induced insulin-resistant rats. Extracts of both 75 or 150 mg/kg doses could decrease the level of glycemia [179]. The decoction extracts of stem bark of *C. pentandra* decreased glucose level by increasing glucose uptake in the liver and skeletal muscle cells by 56.57% and 94.19%, respectively. The extract also reduced the glucose release in liver cells by 33.94% in a hypoglycemic milieu [165]. The ethanolic bark extract of *C. pentandra* at 200 mg/kg dose exhibited antihyperglycemic activity in STZ-induced diabetic rats by decreasing the levels of blood glucose, total cholesterol, and triglycerides, preventing degeneration of liver and pancreas, and increasing serum insulin and liver glycogen content [180]. The aqueous stem bark extracts of *C. pentandra* inhibited alpha-amylase and glucosidase with IC_50_ values of 6.15 and 76.61 μg/mL, respectively, whereas the methanol extract inhibited alpha-amylase and glucosidase with IC_50_ values of 54.52 and 86.49 μg/mL, respectively [168].

### 10.7. Miscellaneous Activities

The petroleum ether and methanol extract from *B. ceiba* stem bark displayed increased osteogenic activity as demonstrated by Chauhan et al. [37] in UMR-106 cells and surgical ovariectomy models in female Wistar albino rats. It has been reported that the administration of the extracts for 28 days ameliorated the consequences of ovariectomy-induced bone porosity, restoring the normal architecture of bone in experimented rats. The in vitro osteogenic activity of the extracts could be attributed to the presence of lupeol, gallic acid, and β-sitosterol in *B. ceiba*.

Komati et al. [163] reported the antiglycation properties of aqueous methanolic calyx extract of *B. ceiba* in methylglyoxal-induced protein glycation and oxidative stress in HEK-293 cells. The extract could inhibit advanced glycation end products (AGEs) formation and restrained Receptor for advanced glycation end products (RAGE) up-regulation in HEK-293 cells.

The aqueous and crude ethanol fruit extracts of *B. ceiba* exhibited diuretic effects in rats. Both aqueous and ethanol extracts could increase the urine output in the rats. The aqueous extract increased the urinary Na+ and K+ levels demonstrating the diuretic effect of the extracts [194]. The ethanolic leaf, bark, and fruit extracts of *A. digitata* exhibited antipyretic activity in albino rats at 400 and 800 mg/kg doses [149].

## 11. Mechanism of Action of Extracts and Bioactive Compounds of the Plants’ Species with Pharmacological Properties

The different plant species of Bombacoideae are well known for their medicinal properties and can act as a useful bio-resource for medicines, nutraceuticals, pharmaceuticals, and chemical analogs for synthetic drugs. Bombacoideae plant species contain several bioactive phytocompounds such as alkaloids, anthocyanins, coumarins, flavonoids, lignans and neolignans, sesquiterpenes, sesquiterpene lactones, sterols, tannins, triterpenes, et cetera, which may be responsible for their antimicrobial properties. The antimicrobial action of phytocompounds might be due to their capacity to disintegrate cytoplasmic membrane, destabilize proton motive force, electron flow, active transport, and coagulation of the cell content in microbes [195]. Silva and Fernandes [196] also reviewed the antimicrobial properties of plants and concluded that different chemical classes of phytochemicals including alkaloids, flavonoids, terpenoids, phenols, tannins, et cetera may be responsible for their antimicrobial potential.

Several phytochemicals of the different classes of compounds such as alkaloids, flavonoids, saponins, terpenoids, vitamins, glycosides, phenols, et cetera play significant roles in inhibiting or arresting cancer cell progression by different mechanisms such as (a) by inhibiting cancer cell-activating signaling pathways such as Cdc2, CDK2, and CDK4 kinases, topoisomerase enzyme, cyclooxygenase, and COX-2, Bcl-2, cytokines, PI3K, Akt, MAPK/ERK, MMP, and TNK; (b) activating mechanisms of DNA repairing, viz. p21, p27, p51, and p53 genes, and Bax, Bid, and Bak proteins; or (c) by stimulating the formation of protective enzymes, viz. Caspase-3, 7, 8, 9, 10, and 12 [197].

Plants enriched with phenolic acids, flavonoids, coumarins, lignans, terpenoids, et cetera can exert antioxidant action by scavenging radicals and chelating metal ions by acting as reducing agents, hydrogen donors, singlet oxygen quenchers, metal chelators, or reductants of ferryl hemoglobin [198]. Therefore, the antioxidant potential of different species of Bombacoideae may be due to the presence of several classes of phytoconstituents including vicenin 2, linarin, saponarin, cosmetin, isovitexin, xanthomicrol, vavain, apigenin, beta-sitosterol, et cetera [151,184].

The different bioactive phytocomponents could exhibit anti-inflammatory activities by down regulating of signaling pathways like NF- κB pathway. This is done by different mechanisms such as (a) inhibiting common mediators of inflammation like NO, iNOS, and pro-inflammatory cytokines like TNF-α, IL-1β, IL-6, and IL-12p40; (b) inhibition of chemokines such as RANTES and MCP-1; (c) downregulating mediators of inflammation such as cycloxygenase-2 (COX-2), prostaglandins, and leukotrienes; (d) reducing the production of ROS and lipid peroxidation; and (e) upregulating enzymatic (superoxide dismutase, catalase, etc.) and non-enzymatic (glutathione, etc.) defense systems [199]. The different species of Bombacoideae such as *A. digitata* and *C*. *pentandra* could inhibit inhibition against proinflammatory cytokine IL-8 expression or by reducing iNOS and NF-*k*B expression [181,182], and the activity is attributed to the presence of different phytoconstituents, viz. quercitrin, cinchonains 1a and 1b, cis-clovamide, trans-clovamide, and glochidioboside [184]. The phytoconstituents of different plants could show antidiabetic activities by inhibiting carbohydrate metabolizing enzymes like amylase and glucosidase enzymes, or by stimulating insulin release or by increasing glucose uptake by cells or by decreasing insulin resistance [200]. Several studies have rightly pointed out that different Bombacoideae plants could exhibit antidiabetic activities by inhibiting α-amylase and α-glucosidase enzymes [148].

## 12. Conclusions

Plants are considered important natural resources as food supplements and in traditional and modern medicine in different regions of the world. Bioactive phytochemicals are valued candidates for the discovery of new drugs. Detailed reporting of plants with food value, and therapeutic and economic importance, of subfamily Bombacoideae, was undertaken in this review. Isolated phytochemicals of diversified classes of secondary metabolites are reported to possess numerous therapeutic properties against different ailments. The bioactive phytochemicals from plants of this subfamily will play important roles in the development of new drug leads with less toxicity and side effects.

## Figures and Tables

**Table 1:** General synopsis of the genera under Bombacoideae.

Genus	Morphological Character	Number of Species	Distribution
*Adansonia* L.	Trunks swollen; leaves simple sometimes lobed; ovary 5–10 locular; fruits indehiscent; 2n = 72, 88, 144, 160. *Adansonia digitata* (2n = 144, 160)	8 species	Mainland Africa, Madagascar introduced to many countries
*Aguiaria* Ducke	Lepidote hairs; leaves simple; staminal tube short with various elongated free filaments; fruits dehiscent; seed ellipsoid	1 species	Amazon region of Brazil
*Bernoullia* Oliv.	Trees leave digitate, staminal tube long, stamens 15–20, fruits dehiscent; seeds numerous, winged	3 species	Mexico to Colombia
*Bombax* L.	Deciduous tree, trunk spiny; leaves digitate; deciduous sepals, fruits dehiscent, seeds winged, determined columella; 2n = 72, 92, 96.	9 species	Tropical Africa, Asia, and Australia
*Camptostemon* Mast. *	Mangrove tree or shrubs; epicalyx fused, enclosing flower; calyx fused; ovary bilocular; fruits dehiscent	3 species	Australia, New Guinea, Borneo, Phillippines
*Catostemma* Benth.	Trees, leaves simple, calyx campanulate; ovary trilocular, fruits dehiscent; cotyledons folded, unequal	15 species	The northern part of South America
*Cavanillesia* Ruiz. & Pav.	Trunks swollen sometimes, leaves simple or palmately lobed, ovary 3–5 locular; fruits winged, indehiscent; 2n = 72, 86, 88	5 species	Panama to Brazil and Peru
*Ceiba* Mill.	Trunks spiny, sometimes swollen; leaves digitate; staminal tube sometimes thickened, stamens 5–15, fruits dehiscent, seeds winged; 2n = 72, 74, 75, 76, 80, 84, 86, 88, 92	21 species	Tropical America, now introduced into the Old World
*Chiranthodendron* Sesse ex Larreat.	Leaves simple to lobed; flowers leaf-opposed; sepals dark red, petals absent, fruits dehiscent	1 species	Mexico, Guatemala
*Eriotheca* Schott & Endl.	Trees unarmed, leaves digitate, staminal tube without phalanges; fruits dehiscent; seeds small, winged; 2n = 92, 210, 270, 6n = 276	23 species	Tropical South America
*Fremontodendron* Coville	Shrubs; leaves simple or lobed, sepals yellow-orange, petals absent, fruits dehiscent	2 species	The southern part of North America
*Gyranthera* Pittier	Tall deciduous tree, leaves digitate, anthers spirally twisted, fruits dehiscent, seeds winged; 2n = 96	2 species	Panama, Venezuela
*Huberodendron* Ducke	Tall trees, hairs stellate, leaves simple; calyx campanulate; fruits dehiscent, seeds winged	3 species	Costa Rica to Brazil, Bolivia, and Peru.
*Lagunaria* (DC.) Rchb. *	Leaves simple, hairs lepidote, epicalyx fused, filaments diverging at different levels; fruits stinging, dehiscent	1 species	Norfolk and Howe Islands, Australia
*Matisia* Humb & Bonpl.	Leaves simple, inflorescences cauliflowers, flowers zygomorphic, fruit drupe	26 species	Tropical America
*Neobuchia* Urb.	Trunk spiny, leaves digitate, stamens 5, anthers twisted; stigmatic branches short; seeds exalbuminous	1 species	Haiti
*Ochroma* Sw.	Tree, leaves simple to lobed, venation palmate; stigma spirally grooved; fruits dehiscent; 2n = 78, 88, 90	1 species	Tropical America
*Pachira* Aubl.	Trunk spiny sometimes; leaves digitate; stamens 90–1000; fruits large, dehiscent, 2n = 72, 82, 88, 92 (neotropical species), 144, 150 (palaeotropical species)	47 species	Tropical Africa, neotropical regions
*Patinoa* Cuatrec.	Trees with verticillate branches, leaves simple, sessile anthers, ovules many, fruits indehiscent	4 species	Colombia to Brazil and Peru;
*Pentaplaris* L.O. Williams & Standl. *	Leaves simple, stipules fused; epicalyx fused, ovary bilocular, fruits indehiscent; cotyledons foliose	3 species	Costa Rica, Ecuador, Bolivia, and Peru
*Phragmotheca* Cuatrec.	Trees, lepidote hairs rare, leaves simple, flowers leaf-opposed; fruit a drupe; cotyledons flat or folded	5 species	Panama to Peru
*Pseudobombax* Dugand	Trunks swollen sometimes, leaves usually digitate, ovary 5 to 8 locular, fruits dehiscent, seeds winged; 2n = 72, 84, 88	22 species	Mexico, Tropical South America
*Quararibea* Aubl.	Trees; lepidote hairs sometimes, calyx usually ridged; ovary 2 to 4 locular, fruit a drupe; n = 72(?)	88 species	Neotropical regions
*Scleronema* Benth.	Tall tree leaves simple, staminal tube short, ovary 2 to 4 locular, fruits dehiscent or indehiscent	3 species	Venezuela, Guyana and Brazil
*Septotheca* Ulbr.	Tall tree, lepidote hairs, simple leaves, cordate, anthers sessile; fruits dehiscent, seeds winged	1 species	Peru, Colombia, and Brazil.
*Spirotheca* Ulbr.	Epiphytic stranglers to the tree, trunk spiny sometimes, leaves digitate, stamens 5, anthers spirally twisted, fruits dehiscent; 2n = 88, 92	5 species	Panama to Peru and Brazil
*Uladendron* Marc.-Berti *	Leaves simple, slightly lobed, fruits dehiscent, seeds winged; cotyledon distorted	1 species	Venezuela

(www.theplantlist.org; accessed on 12 October 2020); * Genera incertae sedis (uncertain placement). Source: (Byng [34]; Fay [36]; Kubitzki and Bayer [8]; Lim [15]; Marinho et al. [35]).

**Table 2:** The main phytochemicals identified in plant species from the Bombacoideae subfamily.

Compound	Plant	Part	Reference
Alkaloids			
Adansonin	*A. digitata*	Seeds and pulp Flowers	[39]
Funebral	*Quararibea funebris*		[40]
Funebradiol			[41]
Funebrine			[42]
***Anthocyanins***			
Cyanidin-3-glucoside	*Ceiba acuminata*		[43]
	*Chorisia speciose* (*Ceiba speciosa* (A.St.-Hil., A.Juss. & Cambess.) Ravenna)		
	*Ochroma lagopus* (*Ochroma pyramidale* (Cav. ex Lam.) Urb.)	Calyx	
	*Pachira aquatica*	Flowers	
Cyanidin-3,5-diglucoside	*Bombax ceiba*		
	*C. speciosa*		
	*Pseudobombax ellipticum*		
	*P. grandiflorum*		
Cyanidin-3-rutinoside	*Pachira aquatica*		
Cyanidin-7-methyl ether-3-β-d-glucoside	*B. ceiba*		[44]
Pelargonidin-5-β-d-glucoside	*B. ceiba*		
Pelargonidin-3,5-diglucoside	*B. ceiba*		[45]
Coumarins			
Cleomiscosine A	*Ochroma lagopus*	Heartwood	[46]
Esculetin	*B. ceiba*	Flowers	[47]
Fraxetin			
Scopoletin			
Scopolin			
Scopoletin	*P. aquatica*	Stems	[48]
Flavonoids			
Apigenin	*B. ceiba*	Flowers	[49]
Apigenin *O*-pentoside	*A. digitata*	Fruits	[50]
Apigenin-7-*O*-β-d-rutinoside	*Chorisia insignis* (*Ceiba insignis* (Kunth) P.E.Gibbs & Semir)	Leaves	[51]
Catechin	*A. digitata*	Fruits	[50]
	*Ceiba pentandra*	Stem bark	[52]
	*Ochroma pyramidale*	Leaves	[53]
Cosmetin	*B. ceiba*	Flowers	[49]
5,4′-Dihydroxy-3,6,7,8-tetramethoxyflavone	*P. aquatica*	Stems	[48]
5,4′-Dihydroxy-3,7-dimethoxyflavone			
Epicatechin	*A. digitata*	Fruits	[50,54]
Epicatechin	*O. pyramidale*	Leaves	[53]
3,5,6,7,8,3′,4′-Heptamethoxyflavone	*P. aquatica*	Stems	[48]
Hesperidin (5,3’-dihydroxy- 4’-methoxy-flavan-7-*O*-α-Lrhamnopyranosyl- (1→ 6)-β-d-lucopyranoside	*B. ceiba*	Roots	[55]
5-Hydroxy-7,4′,5′-trimethoxy isoflavone- 3′-O-α-l-arabinofuranosyl( 1→6)-β-d-glucopyranoside	*Ceiba pentandra*	Stem bark	[56]
5-Hydroxyauranetin	*P. aquatica*	Stems	[48]
5-Hydroxy-7,4’-dimethoxy-flavone	*Bombax anceps*	Roots	[57]
5-Hydroxy-3,7,4’-trimethoxy- flavone	*Bombacopsis glabra* (*Pachira glabra* Pasq.)	Stem bark, root bark	[58]
5-Hydroxy-3,6,7,4’-tetra-methoxyflavone			
5-Hydroxy-3,6,7,8,4’-penta-methoxyflavone			[58]
Isovitexin	*B. ceiba*	Flowers	[49]
Linarin			
3,7-Dihydroxy-flavan-4-one- 5-*O*-β-d-galactopyranosyl- (1→ 4)-β-d-glucopyranoside	*A. digitata*	Roots	[59]
5,7-Dimethoxy-flavone	*Bombax anceps*	Roots	[57]
3,5,7-Trimethoxy-flavone	*B. anceps*	Roots	
Luteolin-7-*O*-β-d-rutinoside	*C. insignis*	Leaves	[51]
Kaempferol	*A. digitata*	Fruits	[50]
	*B. ceiba*	Flowers	[60]
	*C. pentandra*	.	[61]
Kaempferol 3-*O*-galactoside	*A. digitata*	Fruits	[50]
Kaempferol 3-*O*-glucoside	*A. digitata*	Fruits	
Kaempferol 3,7,4′-trimethyl ether	*P. aquatica*	Stems	[48]
Pentandrin	*C. pentandra*	Stem bark	[62]
Pentandrin glucoside	*C. pentandra*	Stem bark	
Quercetin	*B. ceiba*	Flowers	[60]
	*A. digitata*	Fruits	[50]
	*C. pentandra*	-	[61]
Quercetin-3-*O*-glucoside	*A. digitata*	Fruits	[50,54]
Quercetin-7-*O*-xylopyranoside	*A. digitata*	Stem	[63]
Retusin	*P. aquatica*	Stems	[48]
Rhoifolin	*Chorisia crispiflora*	Leaves	[64]
	*Chorisia pubiflora*	Leaves	
	*C. speciosa*	Leaves	[64,65]
Rutin	*A. digitata*	Leaves	[50]
	*C. insignis*	Leaves	[51]
Saponarin	*B. ceiba*	Flowers	[49]
Santin-7-methyl ether	*P. aquatica*	Stems	[48]
Shamimin	*B. ceiba*	Leaves	[66]
Shamimicin	*B. ceiba*	Stem bark	[67]
Tiliroside	*C. speciosa*	Leaves	[65]
Tiliroside isomer	*A. digitata*	Fruits, leaves	[50]
Tiliroside I			
Tiliroside II			
3,3’,4’-Trihydroxy flavan-4-one-7-*O*- α-L-rhamnopyranoside	*A. digitata*	Roots	[54,68]
Vicenin 2	*B. ceiba*	Flowers	[49]
Vitexin	*O. pyramidale*	Leaves	[53]
Xanthomicrol	*B. ceiba*	Flowers	[49]
*Lignans and neolignans*			
Boehmenan	*Ochroma lagopus*	Heart wood	[46]
Boehmenan B	*O. lagopus*	Heart wood	[69]
Boehmenan C			
Boehmenan D			
Bombasin	*B. ceiba*	Flowers	[70]
Bombasin-4-*O*-glucoside			
Bombasinol A			[71]
Carolignan A	*O. lagopus*	Heart wood	[69]
Carolignan B			
Carolignan C			
Carolignan D			
Carolignan E			
Carolignan F			
Dihydro-dehydro-diconiferyl alcohol- 4-*O*-glucopyranoside	*B. ceiba*	Flowers	[70]
5,6-Dihydroxymatairesinol	*B. ceiba*	Flowers	[71]
Matairesinol			
(+)-Pinoresinol			
Secoisolariciresinol diferulate	*O. lagopus*	Heart wood	[46]
*Sesquiterpenes* and *sesquiterpene lactones*			
Aquatidial	*Pachira aquatica*	Root bark	[72]
Bombamalabin	*B. malabaricum*	Root bark	[73]
Bombamalone A		Roots	[74]
Bombamalone B			
Bombamalone C			
Bombamalone D			
Bombamaloside			
7-Hydroxy-cadalene		Roots	[75]
Isohemigossypol-1-methyl ether	*B. anceps*	Roots	[57]
	*B. ceiba*	Root bark	[73,75]
Isohemigossypol-2-methyl ether	*B. anceps*	Roots	[57]
	*B. ceiba*	Roots, root bark	[75,76]
Isohemigossypol-1,2-dimethyl ether			
Isohemigossypol-2,7-dimethyl ether	*B. ceiba*	Roots	[74,75]
Lacinilene C			
Hemigossylic acid lactone-2-hydroxy- 7-methyl ether			
Hemigossylic acid lactone-2-hydroxy- 7-methyl ether	*C. pentandra*	Root bark	[77]
6-Hydroxy-5-isopropyl-3-methyl-7- methoxy-8,1- naphthalene carbolactone	*B. ceiba*	Roots	[78]
Isohemigossylic acid lactone-2- methyl ether	*B. ceiba*	Roots	[74,76]
Isohemigossylic acid lactone-2- methyl ether	*C. pentandra*	Root bark	[77]
5-Isopropyl-3-methyl-2,7-dimethoxy- 8,1-naphthalene carbolactone	*B. ceiba*	Roots	[75]
5-Isopropyl-3-methyl-2,7-dimethoxy- 8,1-naphthalene carbolactone	*C. pentandra*	Root bark	[77]
Sterols			
Campesterol	*A. digitata*	Seeds	[79]
	*B. ceiba*	Flowers	[49]
	*A. fony*	Seeds	[79]
	*A. za*		
	*A. suarezensis*		
	*A. grandidieri*		
	*A. madagascariensis*		
β-Sitosterol	*B. ceiba*	Stem bark	[37]
	*B. ceiba*	Root bark	[73]
	*B. ceiba*	Flowers	[80]
	*A. digitata*	Seeds	[79]
	*C. pentandra*	Stem bark	[62]
Stigmasterol	*A. digitata*	Seed	[79]
	*B. ceiba*	Flowers	[80]
	*A. grandidieri*	Seeds	[79]
	*A. madagascariensis*		
	*A. fony*		
	*A. za*		
	*A. suarezensis*		
Tannins			
Epicatechin-(4β→8)-epicatechin	*A. digitata*	Fruits	[54]
Epicatechin-(4β→6)-epicatechin			
Epicatechin-(2β→O→7, 4β→ 8)-epicatechin			
Epicatechin-(4→β8)-epicatechin- (4→β8)-epicatechin			
Ethyl gallate	*B. ceiba*	Seeds	[81]
Gallic acid		Stem bark	[37]
		Seeds	[81]
1-Galloyl-β-d-glucose			
Tannic acid			
Triterpenes			
β-Amyrin	*C. speciosa*	Leaves	[65]
Lupeol	*B. glabra*	Stem bark, root bark	[58]
	*B. ceiba*	Stem bark	[37]
	*B. malabarica*	Root bark	[73]
	*B. anceps*	Roots	[57]
	*Cavanillesia hylogeiton*	Stem bark	[61]
	*O. pyramidale*	Leaves	[53]
	*P. aquatica*	Root bark	[72]
Oleanolic acid	*B. ceiba*	Roots	[55]
	*O. pyramidale*	Leaves	[53]
Ursolic acid	*A. digitata*	Fruits	[82]
Other compounds			
Argentilactone I	*Chorisia crispiflora*	-	[83]
Argentilactone II	*C. crispiflora*	-	[83]
Bombalin	*B. ceiba*	Flowers	[70]
Bombaxquinone B	*B. anceps*	Roots	[57]
	*B. ceiba*	Roots	[74]
	*C. pentandra*	Root bark	[77]
	*B. ceiba*	Root bark	[84]
(R)-6-[(Z)-1-Heptenyl)]-5,6-dihydro- 2H-pyran-2-one	*C. crispiflora*	-	[83]
Hemigossypolon-6-methyl ether	*B. ceiba*	Root bark	[85]
Isohemigossypolone	*B. glabra*	Stem bark, root bark	[58]
	*B. ceiba*	Root bark	[85]
	*C. pentandra*	Heart wood	[86]
	*P. aquatica*	Root bark	[58,87]
Isohemigossypolone-2-methyl ether	*P. aquatica*	Root bark	[87]
Neochlorogenic acid	*B. ceiba*	Flowers	[70]
*trans*-3-(*p*-Coumaroyl)-quinic acid			
3-Methyl-2(3H)-benzofuranone			

**Table 3:** Plant and parts used as a food.

Name of the Species	Parts Used	Mode of Usage	Country	Reference
*Adansonia digitata* L.	Leaves and seeds	Soup, sauce, fermentation, gruel	Southern Africa, Italy	[15,106]
*Adansonia gregorii* F.Muell.	roots, fruit pulp, seeds, tuberous, young leaves,	Food	Aborigines in Australia	[15]
*Bombax ceiba* L.	Dry cores of the flower	soup	Shan State (Myanmar) and Northern Thailand	[107]
	Flower buds	Vegetable	South India	[108]
	Seeds	Roasted and eaten		[32]
*Bombax costatum* Pellegr. & Vuillet	Unripe fruits and flowers	Soup	Burkina Faso	[109]
*Catostemma fragrans* Benth.	Aril	Fresh	Guianas	[110]
*Cavanillesia platanifolia* (Humb. & Bonpl.) Kunth	Seed, Root	Sweet, water source	Peru	[104,111]
*Ceiba pentandra* (L.) Gaertn.	Young leaves, petals, capsules	Vegetable	Tropical countries of Asia and America, Thailand	[15,32]
*Ceiba aesculifolia* (Kunth) Britten & Baker f.	Young leaves, ripe fruits	Vegetable, Stew	Mexico	[32]
*Pachira glabra* Pasq.	Young Leaves	Vegetable	Equatorial Africa	[32]
*Pachira insignis (Sw.) Savigny*	Seeds, young leaves, flowers	Vegetable	South America	[15]
*Patinoa almirajo* Cuatrec.	Fruit	Edible fruit	Brazil, Colombia	[32]
*Pseudobombax ellipticum* (Kunth) Dugand		Beverage	South America	[33]
*Quararibea cordata (Bonpl.) Vischer*	Fruit	Juice, drinks	South America	[15]
*Quararibea funebris* (La Llave) Vischer	Flowers	Chocolate Drinks, desserts	South America	[33]
	Flowers	Spice	South America	[112]
*Quararibea obliquifolia* (Standl.) Standl.	Fruit	Fresh	Ecuador	[113]

**Table 4:** Economic and traditional uses of Bombacoideae members.

Name of the Species	Parts Used	Purpose	Country	Reference
*Adansonia digitata* L.	Fruit shell	Fuel	Tanzania	[118]
	Leaves	Fodder	The Sahelian region, Africa	[118]
	Fiber from bark	Ropes, textile, basketry, fishing lines	Africa	[118]
	Tree trunk	Reservoir of water	Sudan	[15]
	Roots	Red dye	East Africa	[118]
*Aguiaria excelsa* Ducke	Wood	Boat, construction	Brazil	[28]
*Bombax ceiba* L.	Fiber	Mattress, pillows, cloth	Asia	[117]
*Bombax insigne* Wall.	Wood	Timber, boat construction, matches, plywood	India, Sri Lanka, Nepal	[119,120]
*Bombax costatum* Pellegr. & Vuillet	Wood	Drum, xylophone, match stick, home appliances, door frame, fuelwood	Africa	[109]
	Tannin	Dye	Africa	[109]
	Fruits	Mattress, cushion, pillow	Africa	[109,121]
*Bombax rhodognaphalon* K. Schum.	Leaves, roots	Witchcraft	Africa	[122]
*Catostemma commune* Sandwith	Wood	Timber	Central and Latin America	[123]
*Cavanillesia umbellata* Ruiz & Pav.	Bark	Drum hoops	Peru	[111]
	Wood	Door fillings, light boxes, toothpicks, paper pulps	Peru	[111]
*Ceiba aesculifolia* (Kunth) Britten & Baker f.	Fiber	Fiber	Mexico, Guatemala	[32]
*Ceiba pentandra* (L.) Gaertn.	Fiber, wood	Paper, fiber, insulation material, pillows, toys	Tropical countries	[32,116]
*Ceiba samauma* (Mart. & Zucc.) K.Schum.	Seed	Thermal insulation	Ecuador	[124]
*Ceiba trischistandra* (A.Gray) Bakh.	Fruit wall	Fiber	Java, Peru, and Brazil	[32]
*Huberodendron patinoi* Cuatrec.	Wood	Timber	Colombia	[125]
*Ochroma pyramidale* (Cav. ex Lam.) Urb.	Wood	Bowls, rafts, canoes, toys, carvings (Balsa)	Venezuela	[16]
*Pachira aquatica* Aubl.	Whole tree	Ornamental, fortune tree	East Asia, South East Asia	[15]
*Pachira insignis* (Sw.) Savigny	Wood	Paper	South America	[32]
*Pentaplaris davidsmithii* Dorr & C. Bayer	Wood	Firewood	Bolivia	[126]
*Quararibea funebris* (La Llave) Vischer	Flowers	Perfume	South America	[33]
*Quararibea malacocalyx* A.Robyns & S.Nilsson	Seed fiber	Thermal and acoustic insulation	Ecuador	[124]
*Scleronema micranthum* (Ducke) Ducke	Wood	Construction, joinery, flooring, furniture	Brazil	[127]
*Spirotheca rivieri* (Decne.) Ulbr.	Wood	Box, Linings	Brazil	[128]

**Table 5:** Plants belonging to Bombacoideae with ethnopharmacological uses.

Species	Country	Parts Used	Disease	Mode of Usage	Reference
*Adansonia digitata* L.	India	Pulp	Diarrhea and dysentery	External application	[118]
	India	Leaves	Swellings	Crushed and applied	[118]
	South and East Africa	Leaves	Malaria and fever	Mixed with water	[131]
	Cameroon, Central Africa	Fruits, seeds	Dysentery, fever	Decoction	[131]
	South Africa	Leaves	Diarrhea, fever, kidney and liver diseases, inflammation, asthma	Infusion	[132]
	Nigeria	Bark	Sickle-cell anemia	Aqueous extract	[15,133]
	Burkina Faso	Leaves	Toothache, gingivitis		[134]
*Bernoullia flammea* Oliv.	Guatemala	Seeds	Intoxication	Smoke	[135]
*Bombax ceiba* L.	India	Root	A nocturnal emission, cold, and cough, dysentery, diarrhea, snake bite, gonorrhea, leucorrhea	Drink the powdered solution; applied the paste	[1,136]
	India, Nepal	Bark	Wounds, diarrhea, digestive disorder, heartburn, kidney stone	Paste, Juice	[1,136,137]
	India, Pakistan	Stem, root	Acne, skin blemishes, pimples	Powder	[136,137]
	India, Pakistan	Root	Diabetes		[129,137]
	China	Bark, root	Muscular injury		[137]
	Bangladesh	Seeds, roots	Leprosy		[137]
	India	Fruits	Urolithiasis	Oral administration	[129]
	India	Gum	Asthma, piles, diarrhea and dysentery, dental caries, scabies		[1]
	India	Flower	Hematuria, anemia, leucorrhea, hydrocoele, gonorrhea, menstrual disorders, boils and sores		[1]
*Bombax insigne* Wall.	India	Bark	Dysentery	Tea	[122]
*Bombax buonopozense* P.Beauv.	Africa	Leaves	Venereal disease, constipation, infections		[122]
*Bombax costatum* Pellegr. & Vuillet	Senegal, Sierra Leone, Burkina Faso	Bark	Diuretic properties, dysentery, epilepsy		[109,122]
	Senegal	Leaves	Oedema, snake bite, convulsions, measles	Extract, decoction, paste	[109,121]
*Bombax rhodognaphalon* K. Schum.	Tanzania, Mozambique	Bark	Diarrhea		[122]
*Catostemma fragrans* Benth.	Guianas	Bark	Fever	Decoction	[138]
*Catostemma commune* Sandwith	Guianas	Seed	Snoring		[138]
*Cavanillesia platanifolia* (Humb. & Bonpl.) Kunth	South America, Peru	Bark, oil	Underweight	Infusion	[111,122]
*Ceiba pentandra* (L.) Gaertn.	South America	Immature fruits, roots, leaves barks	Cough, hair shampoo; component of ayahuasca, psychoactive drugs		[15,32]
	Java	Leaves	Intestinal catarrh and urethritis, gonorrhea	Infusion	[15]
	Congo	Bark	Management of sickle cell anemia	Aqueous extracts	[139]
	Philippines	Bark	Vomitive and aphrodiastic	Decoction	[15]
*Ceiba ventricosa* (Nees & Mart.) Ravenna	Brazil		Skin disease, inflammation		[122]
*Chiranthodendron pentadactylon* Larreat.	Mexico	Flowers	Gastrointestinal disorder, diarrhea, dysentery, blood pressure	Infusion	[122]
*Eriotheca globosa* (Aubl.) A.Robyns	South America	Ripe fruits	Cuts, wounds	Application	[122]
*Fremontodendron californicum* (Torr.) Coult.	North America	Bark	Throat irritation	Infusion	[122]
*Huberodendron patinoi* Cuatrec.	Colombia	Bark	Leishmania		[140]
*Huberodendron swietenioides* (Gleason) Ducke	Ecuador	Leaves	Diabetes	Aqueous infusion	[124]
*Matisia glandifera* Planch. & Triana	Colombia	Bark, leaves	Malaria		[141]
*Ochroma pyramidale* (Cav. ex Lam.) Urb.	Brazil	Root bark	Emetic		[142]
*Pachira aquatica* Aubl.	Nicaragua	Bark	Stomach complaint, headache		[15]
*Pachira glabra* Pasq.	India	Leave	Blood pressure, Anemia		[122]
*Pseudobombax ellipticum* (Kunth) Dugand	Guatemala	Bark	Cough and catarrh	Decoction	[143]
*Pseudobombax grandiflorum* (Cav.) A.Robyns	Brazil	Bark	Wound healing	Decoction	[144]
*Quararibea cordata* (Bonpl.) Vischer	South America		Astringent, tonic, antiseptic, for skin infections		[122]
*Quararibea funebris* (La Llave) Vischer	South America	Flowers	Hallucinogenic, psychopathic fears		[40]
*Scleronema micranthum* (Ducke) Ducke	Brazil	Leaf	Toothache		[145]

**Table 6:** Pharmacological studies on some of the plant species of Bombacoideae subfamily.

Plant Species and Part	Part (s) and Solvent	Assay	Results	References
**Antioxidant activity**				
*Adansonia digitata* L.	Methanolic leaf extracts; ethanolic leaf	*In vitro* DPPH, ABTS, FRAP, β-carotene bleaching test, superoxide scavenging assay; CAT and SOD, and GSH assay	The DPPH scavenging activity recorded highest in seed extract (27.69%) and lowest in fruit wall (20.69%) extract. The antioxidant status of the STZ induced diabetic rats are normalized by reducing the elevated levels of reduced glutathione (GSH) superoxide dismutase (SOD), and catalase (CAT)	[50,147,148,149,150]
	Methanolic fruit extracts;	DPPH, ABTS, FRAP assay, β-carotene bleaching test, superoxide scavenging assay	Scavenge the DPPH free radicals with the percentage of inhibition of 13.4, 29.23, and 39.21%, respectively	[50,149]
Bombax malabaricum DC.	*n*-hexane and methanol extracts of flower	DPPH radical scavenging, lipid peroxidation, myeloperoxidase activity	Scavenged DPPH radicals over a concentration range of 0.55–0.0343 mg/mL and 0.5–0.0312 mg/mL, respectively	[49,151]
*Bombax ceiba* L.	Methanolic root; aqueous soluble partitioned of the methanolic root; methanol, dichloromethane, and petroleum ether extracts of roots	The extract exhibited dose-dependent DPPH and reducing power assay. Phenolic constituents donate^.^ OH leading to resonance stabilization	Methanolic root extract could scavenge DPPH radicals, lipid peroxidation, and ascorbyl radicals with an EC_50_ value of 87 µg/mL	[152,153,154,155]
	Aqueous and ethanolic bark Methanolic stem bark	DPPH, ABTS, nitric oxide and superoxide radical scavenging activity, lipid peroxidation, metal chelating, and total antioxidant capacity	Inhibited lipid peroxidation in rat liver microsome induced by ascorbyl and peroxynitrite radicals with an IC_50_ value of 141 µg/mL and 115 µg/mL, respectively	[156,157]
	Methanolic extract of the whole plant	DPPH scavenging assay	IC_50_ values of aqueous extracts of *B. ceiba* varied between 85.71 and 102.45 µg/mL and for ethanolic extract, it varied between 85.48 and 103.4 µg/mL	[158]
	Diethyl ether and light petroleum ether extracts of flowers; Aqueous flower extracts; Methanolic flower extracts	DPPH, metal chelating and beta carotene bleaching test, hydroxyl radical, hydrogen peroxide radical, FRAP assay, reducing power assay	Petroleum ether of *B. ceiba* flowers exhibited DPPH and Fe-chelating activities with IC_50_ values of 37.6 and 33.5 μg/mL and diethyl ether extracts exhibited beta-carotene bleaching test with an IC_50_ value of 58.3 μg/mL.	[80,159,160,161,162]
	the aqueous methanol extract of the calyx	Methylglyoxal induced oxidative stress in HEK-293 cells	Reduced the level of reactive oxygen species (ROS), NADPH oxidase (NOX), and thereby lowered the mitochondrial dysfunction in methylglyoxal induced protein glycation	[163]
*Ceiba pentandra* (L.) Gaertn	seed extracts	DPPH, FRAP, reducing assay, and hydroxyl radical scavenging assay	Decoction, maceration, and methanol scavenged DPPH radical with IC_50_ values of 87.84, 54.77, and 6.15 µg/mL, respectively.	[164]
	Methanol extracts of stem bark; ethyl acetate fraction of stem bark	hydroxyl radical, against lipid peroxidation; DPPH radical scavenging	Scavenge DPPH, nitric oxide, and hydroxyl radicals with IC_50_ values of 27.4, 24.45, and 51.65 µg/mL	[165,166]
	ethanol leaf extract; aqueous and methanol extracts of stem bark	DPPH, nitric oxide, and hydroxyl radical scavenging	The aqueous and methanol stem bark extracts inhibited superoxide (IC_50_ values of 51.81 and 34.26 μg/mL), hydrogen peroxide (44.84 and 1.78 μg/mL) and protein oxidation induced by H_2_O_2_ (120.60 and 140.40 μg/mL).	[167,168]
**Antimicrobial activity**				
*Adansonia digitata* L.	Methanolic, ethanolic leaf, and stem bark extracts	agar well diffusion method		[169]
*Bombax ceiba* L.	Methanolic stem bark	Agar well diffusion method	The order of sensitivity from highest to least was *Staphylococcus aureus > Escherechia coli > Pseudomonas aeruginosa > Bacillus subtilis > Salmonella typhi*	[157]
	Methanolic flower extracts	Agar disc diffusion assay and MIC study.	Exhibited antibacterial activity against *Klebsiella pneumonia, E. coli, P. aeruginosa* (Gram-negative), and *S. aureus, B. subtilis* (Gram-positive) bacteria with the MIC value ranging between 3.125 and 12.500 μg/mL	[38,161]
	methanol, dichloromethane, and petroleum ether extracts of roots	Agar disc diffusion assay	The methanol, dichloromethane, and PE extracts exhibited mild to moderate antibacterial activity against different bacterial strains including *Sarcina lutea, Bacillus megaterium, B. subtilis, S. aureus, B. cereus, P. aeruginosa, Salmonella typhi, E. coli, Vibrio mimicus, Shigella boydii,* and *Shigella dysenteriae* with 7–13 mm zone of inhibition	[155]
Bombax malabaricum DC.	*n*-hexane and methanol extracts of flower	Agar disc diffusion method	n-hexane and methanol extracts (at 100 µg/mL) of demonstrated antimicrobial activities	[49]
*Ceiba pentandra* (L.) Gaertn	Ethyl acetate fraction of leaf and bark; ethanol leaf extract	Agar dilution method	ethyl acetate fraction of leaf and bark of *C. pentandra* showed antimicrobial activity against *E. coli, Salmonella typhi, B. subtilis, Kleibsiella pneumonia*, and *S. aureus*	[167,170]
	aqueous, methanol, ethanol, and acetone extract of seed	Disc diffusion method	dose-dependently inhibits antibacterial activity against *E. coli* and *S. aureus*	[171]
**Anticancer activity**				
*Adansonia digitata* L.	seed and pulp extracts	MTT assay	At 10, 100, and 500 µg/mL dose, the inhibition ranges between 22.57 and 29.96% for MCF-7 cell line; 25.85 and 37.81% for Hep-G2 cell line and 20.75 and 27.34% for COLO-205 cell line. Dichloromethane and methanolic extract demonstrated cytotoxic activity against human bBreast development cell lines BT474 with IC_50_ value of 15.3 ± 0.4 µg/mL	[172]
*Bombax ceiba* L.	diethyl ether and light petroleum ether extracts of flowers	sulforhodamine B (SRB) assay brine shrimp lethality bioassay	Antiproliferative activity against human renal adenocarcinoma cell (ACHN) with respective IC_50_ values of 53.2 and 45.5 μg/mL The petroleum ether, dichloromethane, and methanol extracts of *B. ceiba* roots exhibited cytotoxic effect with LC_50_ values of 22.58, 37.72, and 70.72 μg/mL, respectively	[80]
*Ceiba pentandra* (L.) Gaertn	petroleum and acetone stem bark extracts	Dalton’s lymphoma ascites (DLA or solid tumor) model	At 15 and 30 mg/kg doses could reduce tumor weight by >50% and tumor volume on the 30th day in Dalton’s lymphoma ascites. The petroleum ether, benzene, chloroform, acetone, and ethanolic extract of this plant demonstrated cytotoxicity in a concentration dependent manner after 3 h of incubation with EAC cells with EC_50_values of 53.30, 70.58, 250.48, 67.30, and 56.11 µg/mL, respectively	[173
**Antidiabetic activity**				
*Adansonia digitata* L.	methanolic fruit pulp and leaf extracts	α-glucosidase inhibition assay; α-amylase inhibition assay; STZ induced diabetic rats	IC_50_ values of the fruit extracts ranged between 1.71 *±* 0.23 and 2.39 *±* 0.22 µg/mL while the leaf extract had an IC_50_ value of 1.71 *±* 0.23 µg/mL. Methanolic leaf extract reduced elevated blood glucose, glycosylated hemoglobin levels in streptozotocin (STZ) induced diabetic rats.	[50,148,150]
*Bombax ceiba* L.	dichloromethane, ethanol, and aqueous extracts of thalamus and flower; *n*-hexane fraction of sepal	Alpha-amylase and alpha-glucosidase inhibition assay	The IC_50_ values for alpha amylase inhibition for water extract of thalamus, ethanolic extract of thalamus, ethanolic extract of flower, dichloromethane extract of thalamus, water extract of flower, and dichloromethane extract of flower were 32.95, 33.45, 33.85, 34.95, 35.15, and 35.65 µg/mL, respectively.	[174,175]
	ethanolic root extracts	Alloxan induced diabetic rat	At 400 mg/kg decreased the blood glucose level in diabetic mice	[176]
	Ethanolic leaf extracts	STZ- induced diabetic mice	At 70, 140, and 280 mg/kg doses it decreased fasting blood glucose, glycosylated hemoglobin in diabetic rats	[177]
	Bark extracts	STZ- induced diabetic rats	At 600 mg/kg dose the extract could significantly decrease elevated levels of blood glucose in diabetic rats.	[178]
*Ceiba pentandra* (L.) Gaertn	Aqueous stem bark extracts; aqueous (AE) and methanol (ME) extracts of bark	Dexamethasone-induced insulin resistant rats; STZ- induced diabetic rats; Alpha-amylase and alpha-glucosidase assay	At 75 or 150 mg/kg doses could decrease the level of glycemia in insulin resistant rats. Aqueous stem bark extracts of inhibited alpha-amylase and glucosidase with IC_50_ values of 6.15 and 76.61 μg/mL, respectively, whereas the methanol extract inhibited alpha-amylase and glucosidase with IC_50_ values of 54.52 and 86.49 μg/mL, respectively	[165,168,179,180]
**Anti-inflammatory activity**				
*Adansonia digitata* L.	methanol leaf extracts, aqueous leaf extract	iNOS and NF-*k*B expression in LPS-stimulated RAW264.7 cell	Inhibit NO production with an IC_50_ value of 28.6 µg/mL.	[181]
	fruit pulp extract	inhibition of proinflammatory cytokine IL-8 expression	Leaf extract (70 µg/mL) exhibited better anti-inflammatory activity when compared to pulp extract (247 µg/mL).	[182]
*Bombax ceiba* L.	Petroleum ether, ethanol, and aqueous extracts	HRBC membrane stabilization method.	At 1000 µg/mL concentration exhibited anti-inflammatory potential by stabilizing the HRBC membrane	[183]
*Ceiba pentandra* (L.) Gaertn	ethyl acetate extract of aerial part	MTX-induced nephrotoxic rats	At 400 mg/kg dose could inhibit methotrexate (MTX)-initiated apoptotic and inflammatory cascades	[184]
**Hepatoprotective activity**				
*Adansonia digitata* L.	aqueous extract of fruit; methanolic extract of the fruit	CCL_4_ induced hepatotoxic rats; paracetamol-induced hepatotoxicity in rats	Reduction in serum AST, ALT, ALP, bilirubin levels were observed in carbon tetrachloride (CCL_4_) induced hepatotoxic rat. Level of ALT, AST, ALP, total bilirubin, and total protein measurements were normalized in paracetamol-induced hepatotoxic rats.	[185,186,187,188,189]
*Bombax ceiba* L.	Aqueous flower extracts; Methanolic flower extracts	Histological studies; enzyme assay alkaline phosphates, alanine transaminases, aspartate transaminases, and total bilirubin assay	Decreased elevated levels of glutamic-oxaloacetic transaminase (SGOT), glutamic pyruvic transaminase (SGPT), alkaline phosphatize (ALP), bilirubin, and triglycerides, total protein.	[159,190]
	ethanolic root extracts	Enzyme assay in alloxan induced diabetic mice	At 400 mg/kg decreased the hepatotoxicity in diabetic mice by reducing the elevated levels of SGOT and SGPT	[176]
*Ceiba pentandra* (L.) Gaertn	the methanol extract of stem bark	Enzyme assay paracetamol-induced liver damage in rats	Reduces levels of SGOT, SGPT, ALP, and total bilirubin content.	[191]

## Data Availability

All data presented in the manuscript are available in the form of tables and figures in the manuscript.
